# Development and validation of a machine learning-based nomogram for predicting HLA-B27 expression

**DOI:** 10.1186/s12865-023-00566-z

**Published:** 2023-09-26

**Authors:** Jichong Zhu, Weiming Tan, Xinli Zhan, Qing Lu, Tuo Liang, JieJiang, Hao Li, Chenxing Zhou, Shaofeng Wu, Tianyou Chen, Yuanlin Yao, Shian Liao, Chaojie Yu, Liyi Chen, Chong Liu

**Affiliations:** The First Affiliated Hospital of Guangxi Medical University, Nanning, 530021 P. R. China

**Keywords:** HLA-B27, Machine learning algorithms, Prediction model, Nomogram, Immunological diseases

## Abstract

**Background:**

HLA-B27 positivity is normal in patients undergoing rheumatic diseases. The diagnosis of many diseases requires an HLA-B27 examination.

**Methods:**

This study screened totally 1503 patients who underwent HLA-B27 examination, liver/kidney function tests, and complete blood routine examination in First Affiliated Hospital of Guangxi Medical University. The training cohort included 509 cases with HLA-B27 positivity whereas 611 with HLA-B27 negativity. In addition, validation cohort included 147 cases with HLA-B27 positivity whereas 236 with HLA-B27 negativity. In this study, 3 ML approaches, namely, LASSO, support vector machine (SVM) recursive feature elimination and random forest, were adopted for screening feature variables. Subsequently, to acquire the prediction model, the intersection was selected. Finally, differences among 148 cases with HLA-B27 positivity and negativity suffering from ankylosing spondylitis (AS) were investigated.

**Results:**

Six factors, namely red blood cell count, human major compatibility complex, mean platelet volume, albumin/globulin ratio (ALB/GLB), prealbumin, and bicarbonate radical, were chosen with the aim of constructing the diagnostic nomogram using ML methods. For training queue, nomogram curve exhibited the value of area under the curve (AUC) of 0.8254496, and C-value of the model was 0.825. Moreover, nomogram C-value of the validation queue was 0.853, and the AUC value was 0.852675. Furthermore, a significant decrease in the ALB/GLB was noted among cases with HLA-B27 positivity and AS cases.

**Conclusion:**

To conclude, the proposed ML model can effectively predict HLA-B27 and help doctors in the diagnosis of various immune diseases.

**Supplementary Information:**

The online version contains supplementary material available at 10.1186/s12865-023-00566-z.

## Introduction

Human leukocyte antigen (HLA) class I molecule B27 (HLA-B27) is commonly expressed in patients suffering from rheumatoid inflammatory diseases and often shows association with the growth of spondyloarthritides (SpA) [1]. HLA-B27 can be frequently used in the clinical diagnosis of ankylosing spondylitis (AS). HLA-B27 is positive in more than 90% of AS patients. Of course, the diagnosis of AS should also be combined with the clinical symptoms and imaging data of patients [2]. Studies have confirmed that HLA-B27-associated extraocular disease occurs in three quarters of B27-associated uveitis cases. Uveitis is often the first indication for previously undiagnosed HLA-B27-associated extraocular disease [3]. HLA-B27 positivity is closely associated with the recurrence of juvenile arthritis, reactive arthritis, herpetic eye disease and chronic periarteritis [3, 4].

Encoded on chromosome 6, HLA builds major compatibility complex (MHC) in human being, which is the protein with the highest polymorphism at present. B27 allele is regarded as a normal B allele within humans. HLA-B27 mainly functions to deliver endogenous peptides into T lymphocytes [5]. In addition, pathogenesis of HLA-B27 remains unclear, and various theories have been proposed [6]. For example, the pathogenesis of HLA-B27 in AS is related to the ganglogenic peptide hypothesis, HLA-B27 misfolding and homodimer [1, 7]. It has been demonstrated that HLA-B27 homodimer can induce uveitis through innate immune mechanism secondary to killer immunoglobulin-like receptor tuberculosis. Intestinal microbial peptides and HLA-B27-derived peptides lead to the development of uveitis [8].

Machine learning (ML) refers to a scientific subject illustrating the way by which computers learn from information. Besides, ML represents statistics intersection, including data-based relationships exploration as well as computer science, and it highlights effective algorithms for computation. At present, ML has been extensively applied to investigate associated data in clinic, ML methods are flexible and diverse, and different ML can be applied to different clinical data [9, 10]. ML is divided into supervised learning, unsupervised learning and reinforcement learning. This study aimed to investigate the differences between HLA-B27 negatives and positives using multiple ML methods for supervised learning. ML is used to derive prediction functions from labeled training data.

In this study, the intersection method has been widely used in papers related to bioinformation analysis, which is used to take the intersection of different genome expressions [11]. Different ML methods have different advantages in data screening. More and more methods of using multiple ML to obtain specific intersection and build models are applied in clinical practice. Our team has used a similar method to compare the differences between the blood of AS patients and the general population [12]. Han et al. used a similar approach to search for biomarkers of glomerular injury in diabetic nephropathy [13].

We found that many regional hospitals lack the testing equipment for HLA-B27, which affects the diagnosis. We aimed to determine the differences by collecting clinical data related to HLA-B27 test patients and establish a diagnostic model to infer the results of HLA-B27 by using ML methods to help clinicians better diagnose diseases. To enrich our research content and gain a better understanding of the significance of differences in HLA-B27 outcomes among AS patients, we have utilized the available data of AS patients to analyze the variations in clinical symptoms between HLA-B27-positive and negative AS individuals.

## Materials

First Affiliated Hospital of Guangxi Medical University was responsible for flow cytometry (FCM, IOTest®HLA-B27 FITC/HLA-B7-PE, Immunotech S.A.S(a Beckman Coulter Company; 130 Avenue de Lattre de Tassigny, BP 17713276 Marseille Cedex 9, France) was conducted to identify HLA-B27 at clinical laboratory. The assay was conducted according to binding ability of certain monoclonal antibodies (mAbs) to epitopes denoted on surface of leukocytes.

## Patients and methods

In the study, informed consents were obtained from volunteers participating. Approval of this work was acquired from Ethics Committee of First Affiliated Hospital of Guangxi Medical University (NO.2022-KY-E-(211)).

From 2012 to 2021, we screened 1503 patients from First Affiliated Hospital of Guangxi Medical University by performing HLA-B27 examination along with complete C-reactive protein (CRP), erythrocyte sedimentation rate (ESR), liver function test, kidney function test, and blood routine examination. Inclusion criteria: (1) Patients who underwent HLA-B27 examination in the hospital. (2) The patient has complete blood routine, liver and kidney function and other blood test results. (3) Patients volunteered to participate in the study and signed the consent form. Patients under 16 years of age were notified to their families and their family members signed their consent to participate in the study. Exclusion criteria: (1) Patients had participated in multiple HLA-B27 examinations with inconsistent results. (2) Partial blood test results were missing. (3) The patient had been found to have serious cardiovascular, cerebrovascular, liver and kidney diseases before examination. (4) Patients did not agree to participate in the study.

The patient data were anonymized. We employed a probability-based random sampling method with a ratio of approximately 7:3 to randomly allocate the training and validation sets. The training cohort consisted of 509 cases with HLA-B27 positivity, whereas 611 with HLA-B27 negativity. Validation cohort included 147 cases with HLA-B27 positivity while 236 HLA-B27 with negativity.

This work acquired the clinical information in digital records from First Affiliated Hospital of Guangxi Medical University. Data regarding the age, sex, CRP, ESR, liver/kidney function tests, and blood routine examination of each case were gathered and investigated in a statistical way. To be specific, blood routine examination contained red blood cell (RBC) count, white blood cell (WBC) count, blood platelet count (BPC), platelet distribution width (PDW), mean platelet volume (MPV), hematocrit value (HCT), hemoglobin (HGB), mean corpuscular hemoglobin (MCH), mean corpuscular volume (MCV), mean corpuscular hemoglobin concentration (MCHC), percentage of neutrophils (NEUT), absolute value of neutrophil (NEUT#), percentage of lymphocytes (LYM), absolute value of lymphocytes (LYM#),percentage of monocytes (MONO), monocyte absolute value (MONO#), percentage of eosinophils (ESO), absolute value of eosinophils (ESO#), percentage of basophils (BASO), absolute value of basophils (BASO#), thrombocytocrit (PCT) as well as red cell distribution width (RDW). In addition, liver function tests included total bilirubin (TBil), indirect bilirubin (IBil), direct bilirubin (DBil), DBil/IBil, albumin (ALB), globulin (GLB), ALB/GLB ratio, total protein (TP), total bile acid (TBA), gamma-glutamyl transpeptidase (GGT), alanine aminotransferase (ALT), aspartate aminotransferase (AST), AST/ALT, A-alkaline phosphatase (ALP), cholinesterase (ChE) and prealbumin (PAB). In addition, kidney function tests included creatinine (Cr), blood urea nitrogen (BUN), bicarbonate radical (HCO), uric acid (UA), cysteine C (Cys-C) as well as creatinine clearance rate (Ccr).

Due to the modification in the hospital's inpatient case system, we are only able to collect detailed inpatient data from 2018 onwards. All 148 patients with AS were inpatients from Department of Spinal Surgery of First Affiliated Hospital of Guangxi Medical University during 2018–2021. Following New York criteria after modification (Evaluation of AS diagnostic criteria), data regarding sex, age, ALB/GLB ratio, and other information of the patients were gathered through the electronic system of the hospital. Bath Ankylosing Spondylitis Disease Activity Index (BASDAI) score and Bath Ankylosing Spondylitis Functional Index (BASFI) score, and information regarding morning stiffness and night pain were collected through questionnaires. There were 124 cases with HLA-B27 positivity, whereas 24 with HLA-B27 negativity suffering from AS.

## Statistical analysis

IBM SPSS Statistics 23 software was employed to perform data analysis. We adopted student *t-*test to compare means of continuous factors among the patients (cases with HLA-B27 positivity and those with HLA-B27 negativity), Normal distribution and homogeneity of variance were checked before use. Chi-square test was utilized to explore CRP, sex, together with symptoms of patients. SPSS indicated the diverse factors. *P* < 0.05 upon two-tailed probability test indicated statistical significance.

R software (v. 4.1.3; https://www.R-project.org) was employed for statistical analysis. Based on “rms” package, the AS forecasting was accomplished with nomogram survival model, and the nomogram predictability was validated through single-variable logistic regression and C-value estimation [14, 15]. For nomogram prediction assessment, Harrell’s concordance index and AUC were utilized. The nomogram discrimination was examined with Harrell’s concordance index through a bootstrap procedure involving 1,000 samples [16]. The clinical practicality of nonadherence nomogram was evaluated by analyzing decision curve, where the net benefits were measured at varying threshold probabilities among the patients positive with HLA-B27 [17]. Acquisition and visualization of the thresholds were accomplished via the “rms” plus “rmda” packages. We constructed the correlation plot using the corrplot package and calculated the correlation coefficients between two variables using the cor function. The correlation coefficients range from -1 to 1, where positive and negative values indicate the direction of the correlation, and the absolute values represent the strength of the correlation.

In this study, because of the number of patients and the number of variables. We would have tried to use ML directly to look for differences, but our computers were unable to calculate such large numbers. Therefore, we first used SPSS software to screen the data and then carried out the next research.

## Random forest

Randomforest (RF) is a compositional supervised learning method. The RF model can be understood as a decision tree model embedded in the bagging framework. Firstly, the parameters of bagging framework in outer layer are optimized, and then the parameters of decision tree model in inner layer are optimized. The variables in the present work were screened via the R’s “randomforest” package, followed by estimation and visualization of their relative importance [18]. We take advantage of the "varlmp" function in the RF importance ranking. The mean squared error elevation was denoted by “%IncMSE”. Every predictive parameter was assigned with a value stochastically, with a greater value indicating higher parameter importance [19]. Determination of “IncNodePurity”, which stands for the node purity elevation, was accomplished by the residual sum of squares [20], with a greater value also indicating higher parameter importance. The predictive parameter importance was evaluated by either %IncMSE or IncNodePurity [21]. Applying 5 times tenfold cross-validation, the quantity of highest importance was acquired as the most appropriate predictive parameter. We used the "ggplot2" function to draw the fitting line graph.

### Support Vector Machine Recursive Feature Elimination (SVM-RFE)

By adopting “e071” package, the AS forecasting was accomplished in the present research by modeling the SVM-RFE, the potent ML tool. Data were subjected to tenfold cross-validation, the output vector characteristic index was derived, followed by sorting of the parameters from least to most usefulness. A less value of AvgRank indicated that the independent variable was more tremendously impacted by the dependent variable. Upon completion of the sorting, generalization of error estimation proceeded on all of the data, and a subsequent screening of the variable whose common diagnostic error rate was the lowest [22].

## LASSO regression

The degree of complexity adjustment of LASSO regression is controlled by the inclusion of λ. The greater the λ, the greater the punishment for the linear model with more variables, so as to obtain a model with smaller variables. As a contraction approach, LASSO regression actively chooses the huge, possibly multicollinear parameter set for screening optimal predictive traits and risk factors from the patients’ data [23]. Our present work utilized dependent variables that conformed to *P* < 0.05 following Student’s *t*-test computation. Meanwhile, LASSO regression and visualization were carried out via the R’s “glmnet” package [24].

## Results

### Basic data

Tables 1 and 2 present a summary of differences in ESR, CRP, liver/kidney function tests and blood routine examination results of cases with HLA-B27 positivity and those with HLA-B27 negativity in both cohorts. According to Table 1, male proportion in both training cohort and the validation cohort was greater than that of females; moreover, the difference in the number of males and females in the training cohort was statistically significant. In the training and validation cohorts, the average age of HLA-B27 negative patients is higher than that of HLA-B27 positive patients. However, the differences in age and gender in the validation cohort did not reach statistical significance, possibly due to the random assignment of subjects. In the training cohort and the validation cohort it was shown that CRP ratio among cases with HLA-B27 positivity within the range of 10.1–139.9 was considerably higher in relative to that in those with HLA-B27 negativity, and that negative patients occupied the majority in the range of CRP < 10. In the training and validation cohort, the average age, MCV, MCH, MCHC, MPV, and LYM of cases with HLA-B27 positivity were lower compared with patients with HLA-B27 negativity and were statistically significant. Furthermore, the ESR, WBC, RBC, BPC, PDW, NEUT, NEUT#, MONO#, ESO, RDW, and PCT among patients with HLA-B27 positivity markedly increased in relative to patients with HLA-B27 negativity in the two cohort. However, there existed no obvious difference in HGB and other variables between the training cohort and the validation cohort.

**Table 1:**
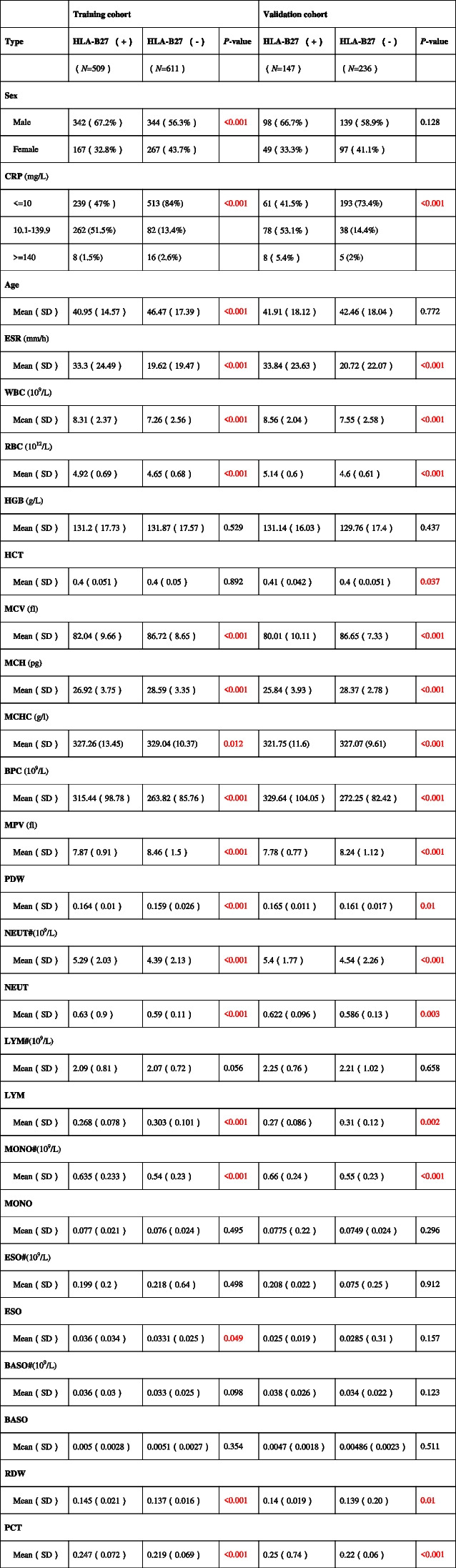
The differences blood routine examination and CRP

The red text means that the *p* value was < 0.05. # denotes absolute value. “ + ” means positive and “-” means negative

**Table 2:**
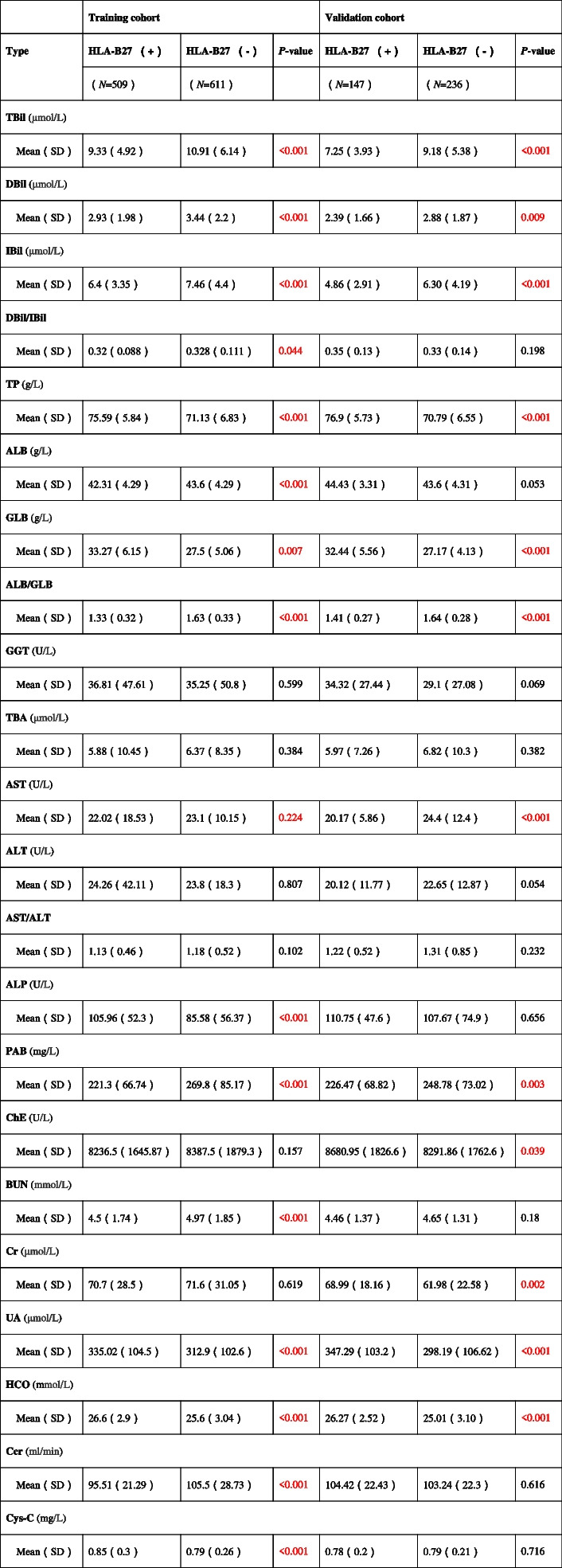
The differences of liver function examination and kidney function examination

The red text means that the *p* value was < 0.05

According to Table 2, in the training cohort, the TBil, DBil, IBil, DBil/IBil, ALB, ALB/GLB ratio, AST, and PAB of cases with HLA-B27 positivity decreased relative to those of cases with HLA-B27 negativity. Opposite differences were observed in the DBil/IBil and ALB ratios in the validation cohort, but these differences were not statistically significant. This discrepancy may be attributed to sampling errors. In two cohort, TP, GLB, and ALP among patients with HLA-B27 positivity increased in comparison with patients with HLA-B27 positivity. There existed no statistical difference in other variables.

It can be seen from Table 2 that in the training cohort, the BUN and Ccr of cases with HLA-B27 positivity were lower when relative to those of HLA-B27 negativity. There was no statistically significant difference observed between them in the validation cohort. In the training cohort, UA, HCO, and Cys-C were higher in HLA-B27-negative patients. But there was no statistically significant difference observed between Cys-C levels in the validation cohort. Moreover, there existed no statistical visible difference in Cr between the two cohorts.

**Fig. 1:**
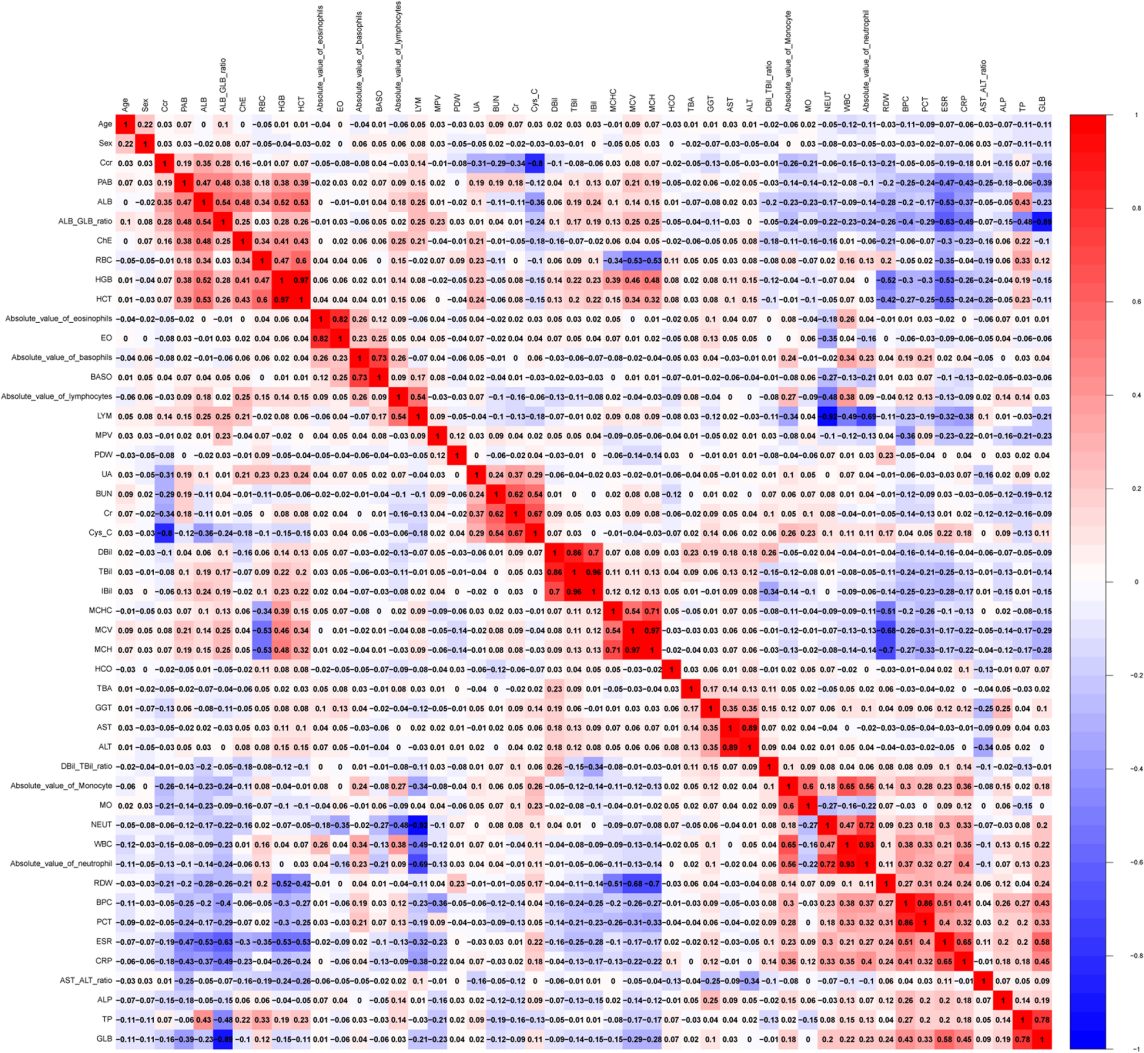
Heatmap showing associations of diverse involved variables are shown

The correlation between all the variables of the training and validation cohorts is illustrated in Fig. 1. HGB and HCT, BASO, absolute basophils, EO and absolute eosinophils, BUN and Cr, Cys-C and Cr, DBil and TBil, TBil and IBil, DBil and IBil, MCV and MCH, MCHC and MCH, AST and ALT, WBC and NEUT, NEUT and the absolute neutrophils, TP and GLB, as well as BPC and PCT exhibited a positive correlation, whereas Cys and Ccr, NEUT and LYM, RDW and MCV, RDW and MCH, and GLB and ALB/GLB ratio exhibited a negative correlation.

### Machine learning

After preliminary screening by SPSS, the variables with *P* < 0.05 were selected, and then ML was used to further screen the variables.

### Random forest

**Fig. 2:**
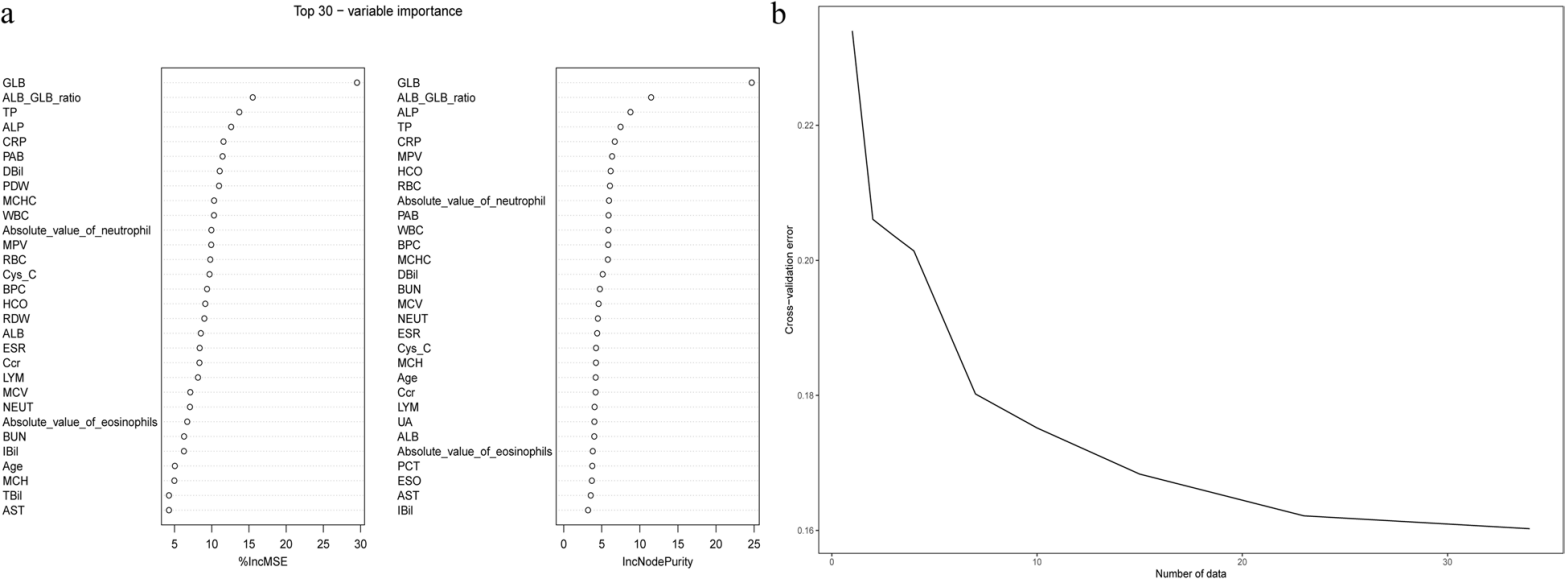
Randomforest screening variables. **a** The 30 most vital elements measured based on the 2 random forest algorithms including “IncNodePurity” and “%IncMSE”. **b** This perfect regression impact is acquired through maintaining 20 vital variables following 10-fold cross-validation

Figure 2a shows the top 30 variables screened using the RF methods of “%IncMSE” and “IncNodePurity”. Figure 2b shows that using RF for selecting 20 variables for diagnosis yielded the best performance. The top 20 variables (Table 3) were screened by combining the %IncMSE and IncNodePurity methods.

**Table 3:** The top 20 variables are screened after combining "%IncMSE" and "IncNodePurity"

Type	%IncMSE	IncNodePurity	Type	%IncMSE	IncNodePurity
GLB	29.528091	24.689556	WBC	10.300806	5.874292
ALB/GLB	15.510579	11.479635	BPC	9.368241	5.840838
ALP	12.608679	8.768030	MCHC	10.315155	5.801214
TP	13.707016	7.469052	DBil	11.072646	5.122757
CRP	11.578667	6.706310	BUN	6.273594	4.754315
MPV	9.930096	6.357899	MCV	7.115748	4.583968
HCO	9.131109	6.168996	NEUT	7.071780	4.488072
RBC	9.802478	6.075653	ESR	8.378661	4.398344
NEUT#	9.951013	5.949444	Cys-C	9.704187	4.253772
PAB	11.458047	5.889966	MCH	4.973804	4.240079

## LASSO

**Fig. 3:**
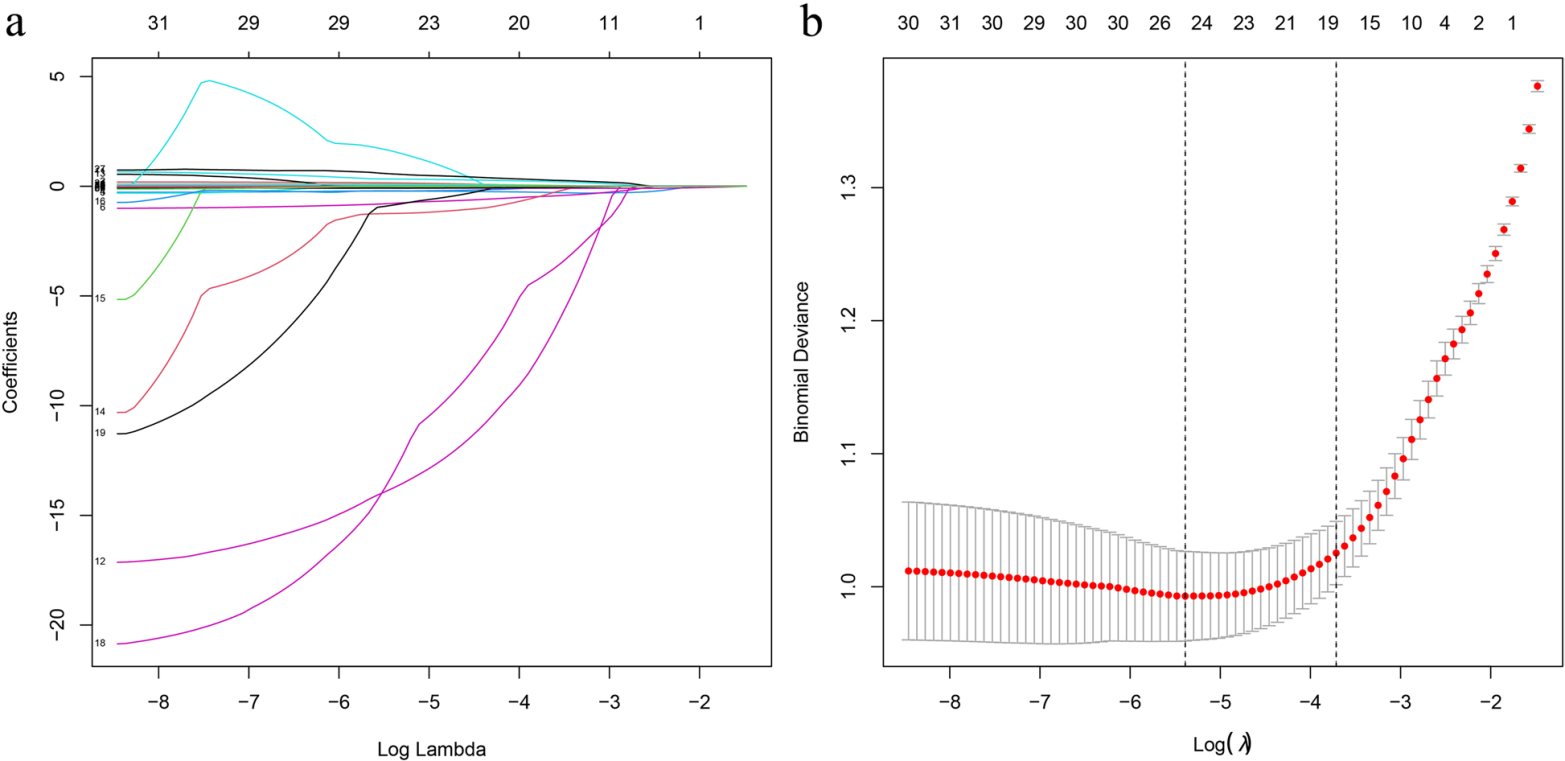
Cross-validation was used for the LASSO coefficient profiles and optimum penalty parameter lambda for factors. **a** LASSO regression for dependent variables. **b** 24 significantly different variables in cases with HLA-B27 positivity relative to negativity

LASSO regression for dependent variables is presented in Fig. 3a. Figure 3b presents 24 significantly different variables between cases with HLA-B27 positivity and those with HLA-B27 negativity.

## SVM-RFE

**Fig. 4:**
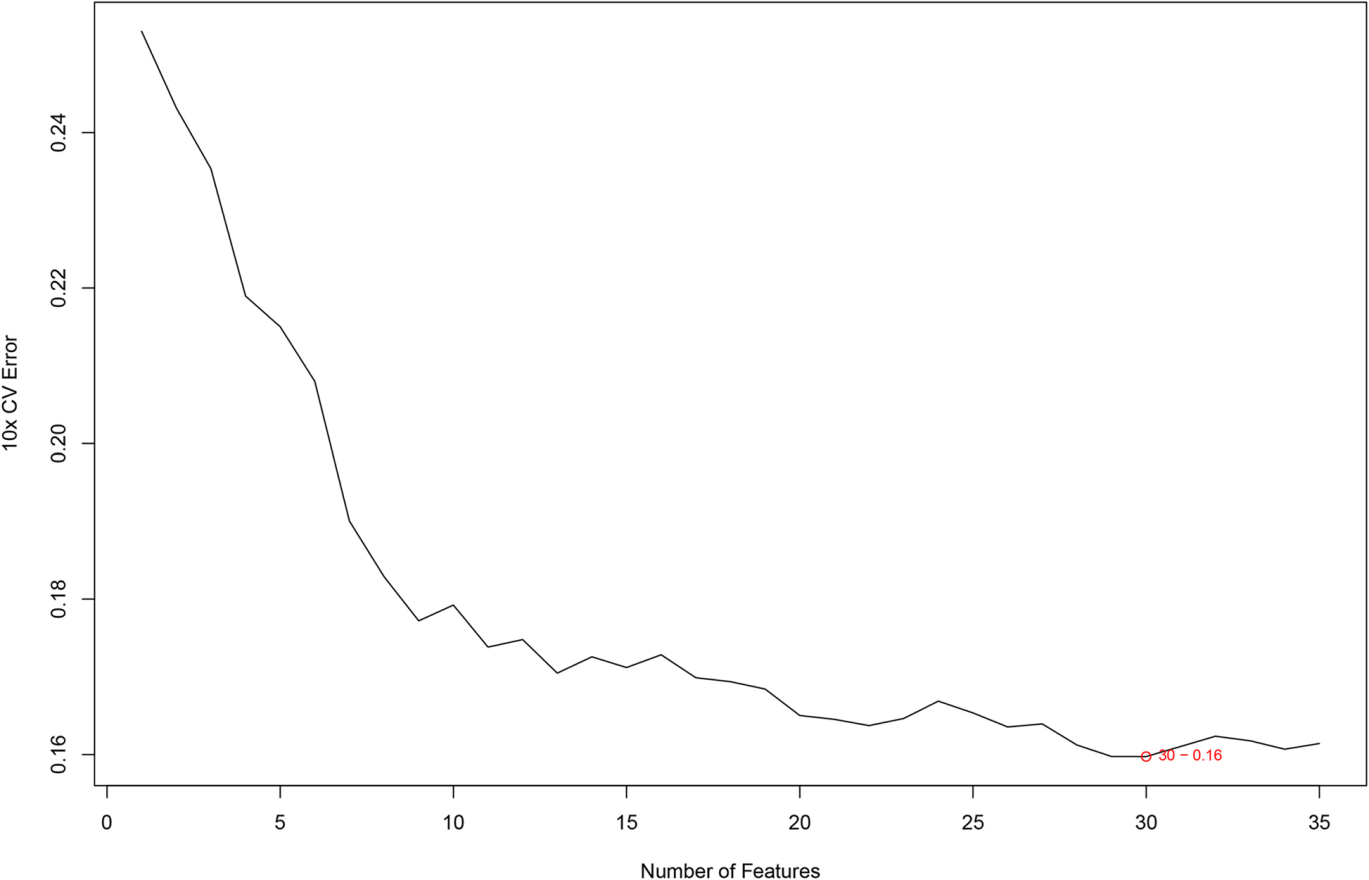
Thirty factors were chosen to be diagnostic models using SVM-RFE calculation

With 30 variables being chosen to be model for diagnosis following SVM-RFE analysis, the minimum error rate was reached, with diverse variables involved being significant in diagnosis (Fig. 4). Table 4 lists the order of importance of There were 30 factors obtained from SVM-RFE, which were displayed by the importance degree in Table 4. It indicates that the reduced factor AvgRank indicated the more importance.

**Table 4:** Importance ranking of 30 variables for SVM-RFE

Type	AvgRank	Type	AvgRank	Type	AvgRank
NEUT	5.1	HCO	12.5	PDW	19.7
MPV	5.6	MCHC	13.1	BPC	20.6
RBC	7.6	ALB/GLB	14	BUN	21.0
MONO#	8.0	DBil/TBil	14.5	MCH	23.1
PAB	8.1	Ccr	16.8	GLB	23.4
NEUT#	9.2	UA	17.3	CRP	23.5
LYM	9.7	MCV	17.4	TBil	23.5
Age	11.5	Cys-C	17.6	TP	24.6
ESO	11.6	PCT	18.8	ALB	25.1
RDW	12.5	IBil	19.3	Sex	26.6

**Fig. 5:**
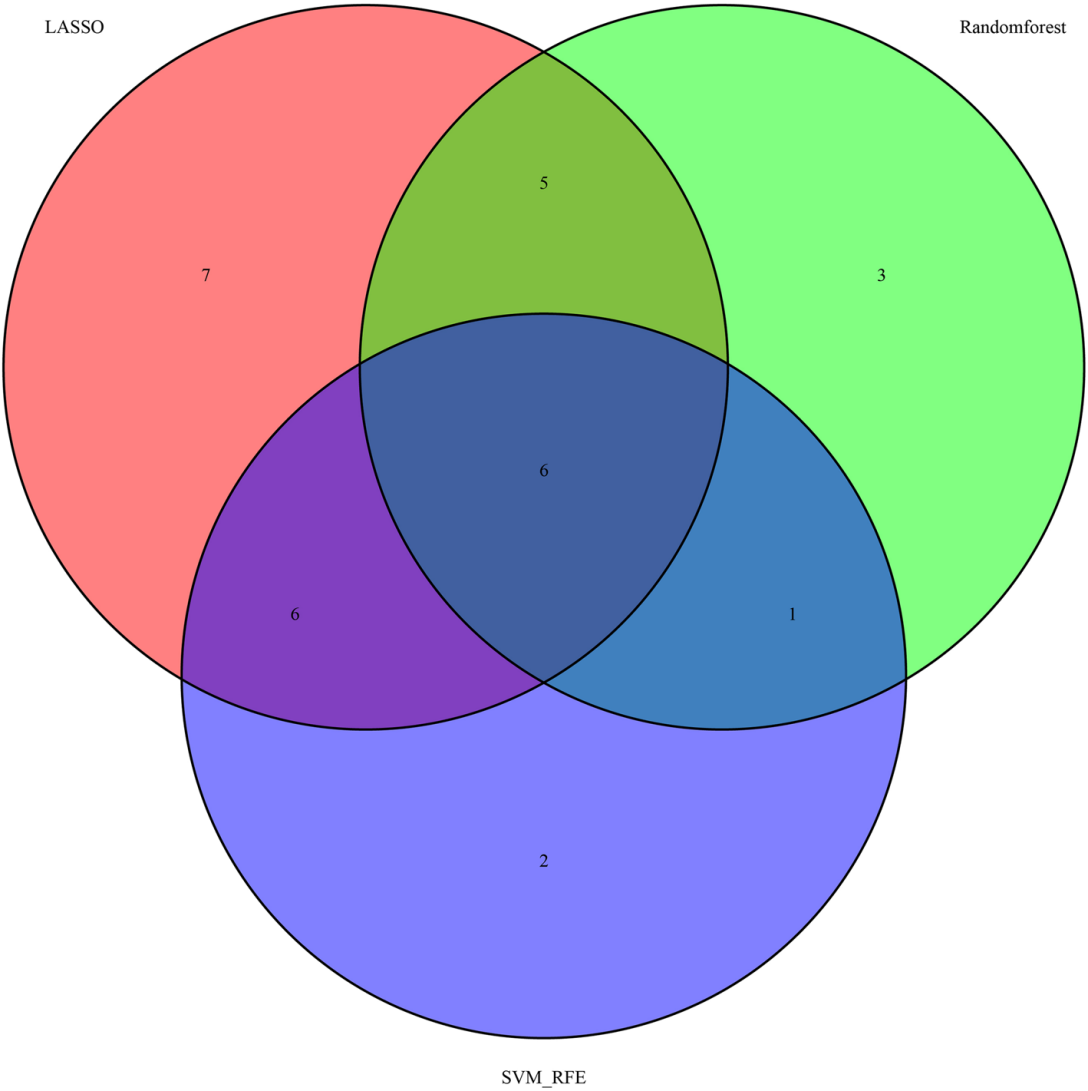
Selected variable intersection based on LASSO, SVM-RFE as well as Random forest

**Fig. 6:**
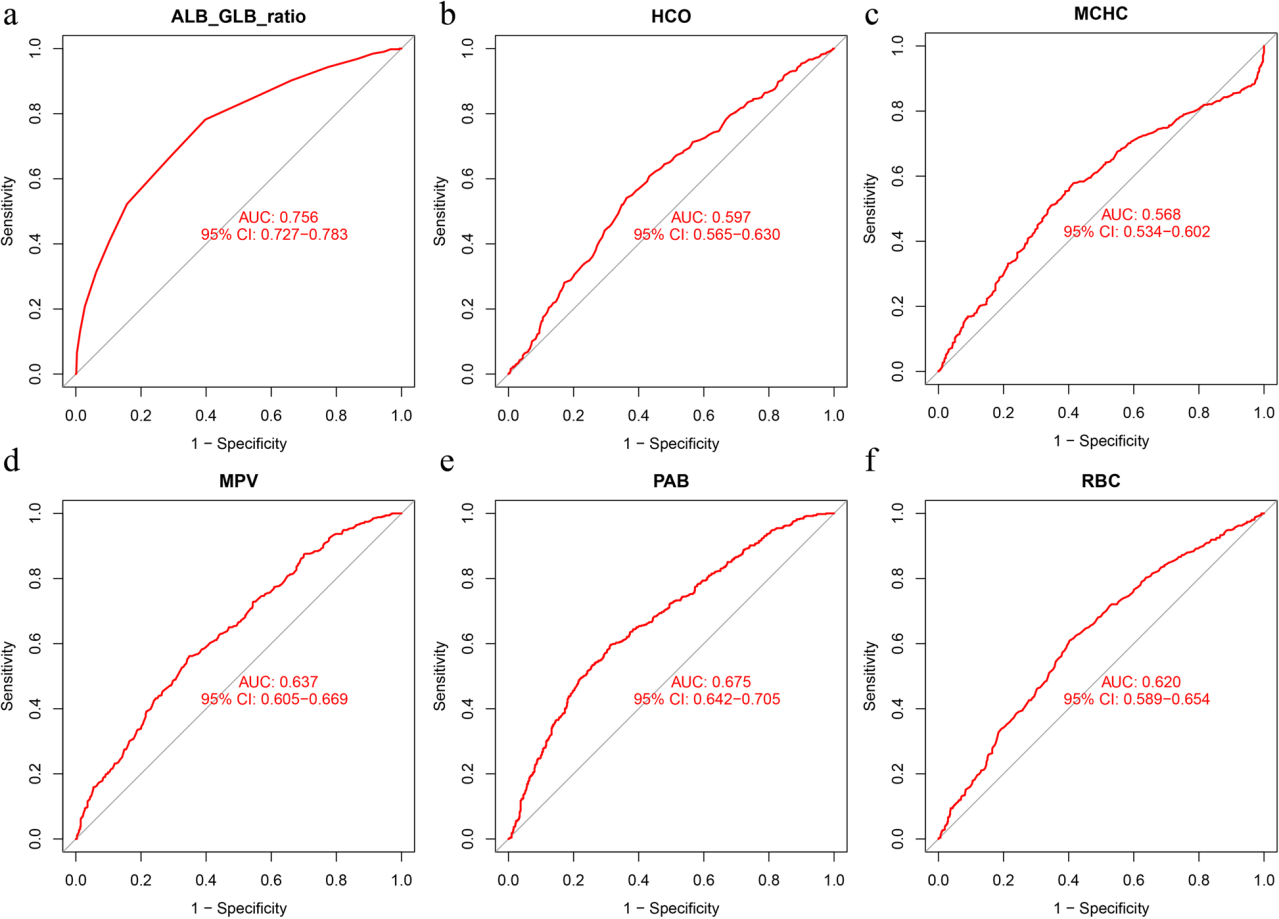
AUCs for selected variable intersection

RF and SVM-RFE were used to screen the top 20 factors and the intersection of LASSO regression analysis results; finally, six factors, namely ALB/GLB ratio, HCO, MCHC, MPV, PAB, and RBC, were obtained for the proposed diagnostic model (Fig. 5). Figure 6a–f shows areas under ROC curve (AUC) for 6 variables.

**Fig. 7:**
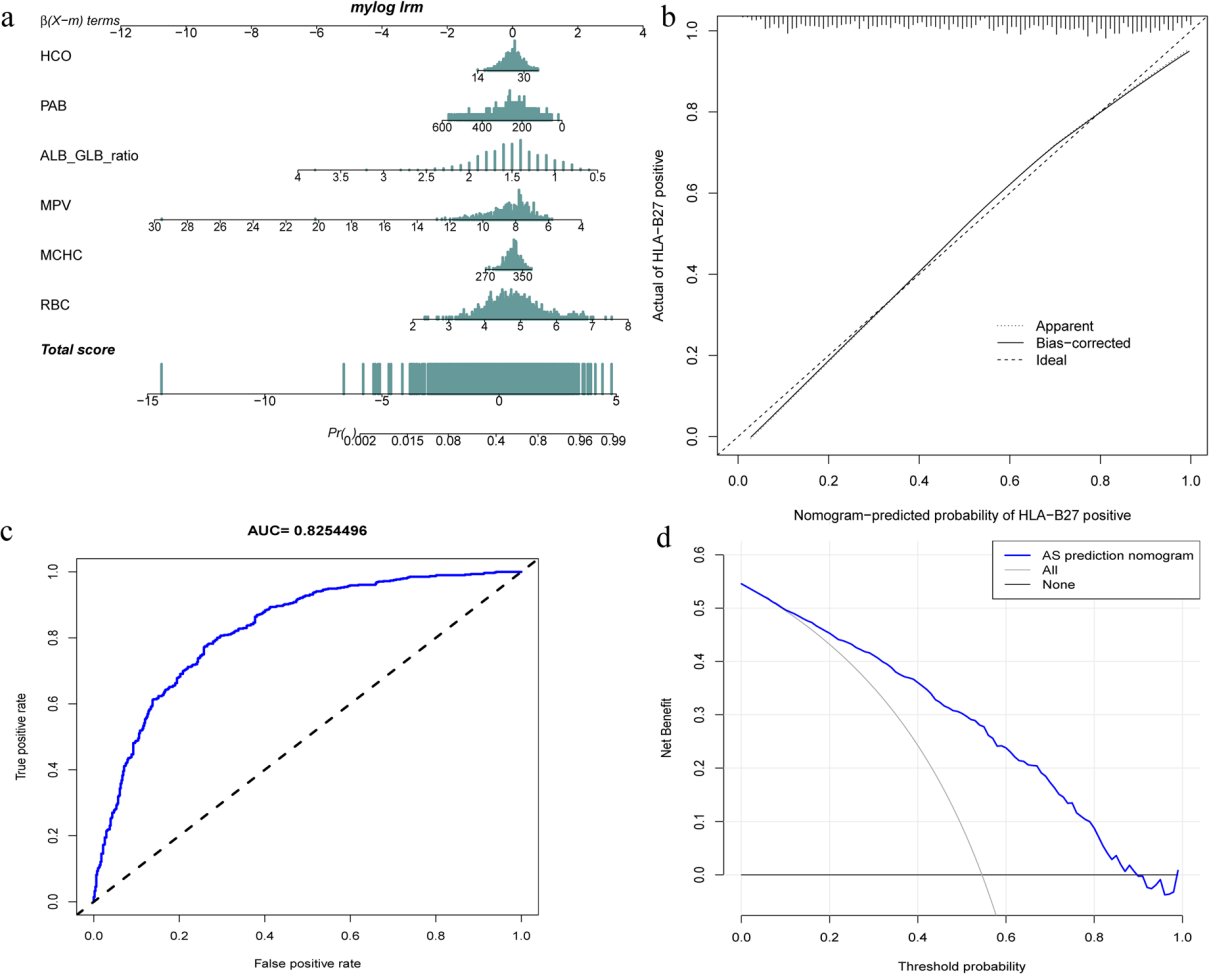
The HLA-B27 model for training cohort. **a** HLA-B27 probability predicted by the nomogram. **b** Calibration curves for prediction of HLA-B27 probability. **c** Nomogram AUC based on HLA-B27 diagnostic model. **d** Decision curve analysis showing HLA-B27 model

The nomogram model constructed using the six aforementioned factors is depicted in Fig. 7a. According to the calibration curves, the nomogram predicted values were consistent with real measurements (Fig. 7b). Obviously, C-value of the model was 0.825, and the nomogram curve attained the AUC value of 0.8254496 (Fig. 7c). The decision curve shown in Fig. 7d showed that with the model threshold being set within 2%–90%, the decision curve could be detected above ALL and NONE lines, suggesting the model’s clinical utility within the current scope.

**Fig. 8:**
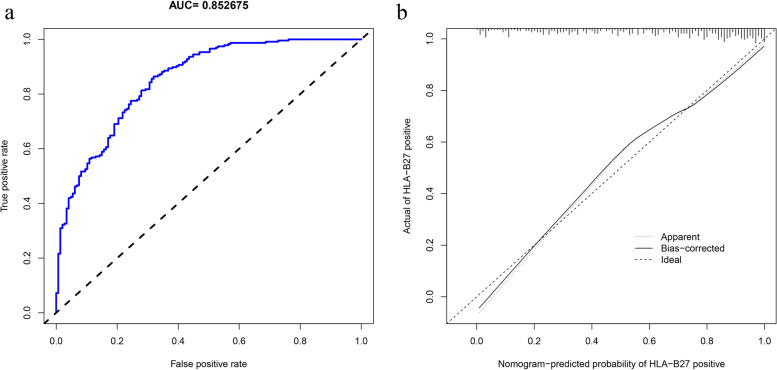
Validation cohort. **a** The AUC value of HLA-B27 diagnostic model in the validation cohort. **b** Calibration curves of HLA-B27 model for validation set

The proposed model was used for the validation cohort, and its AUC and C-value were calculated as 0.852675 and 0.853, respectively (Fig. 8a). According to the calibration curve, nomogram predicted value was consistent with real measurement (Fig. 8b).

### ALB/GLB ratio

**Fig. 9:**
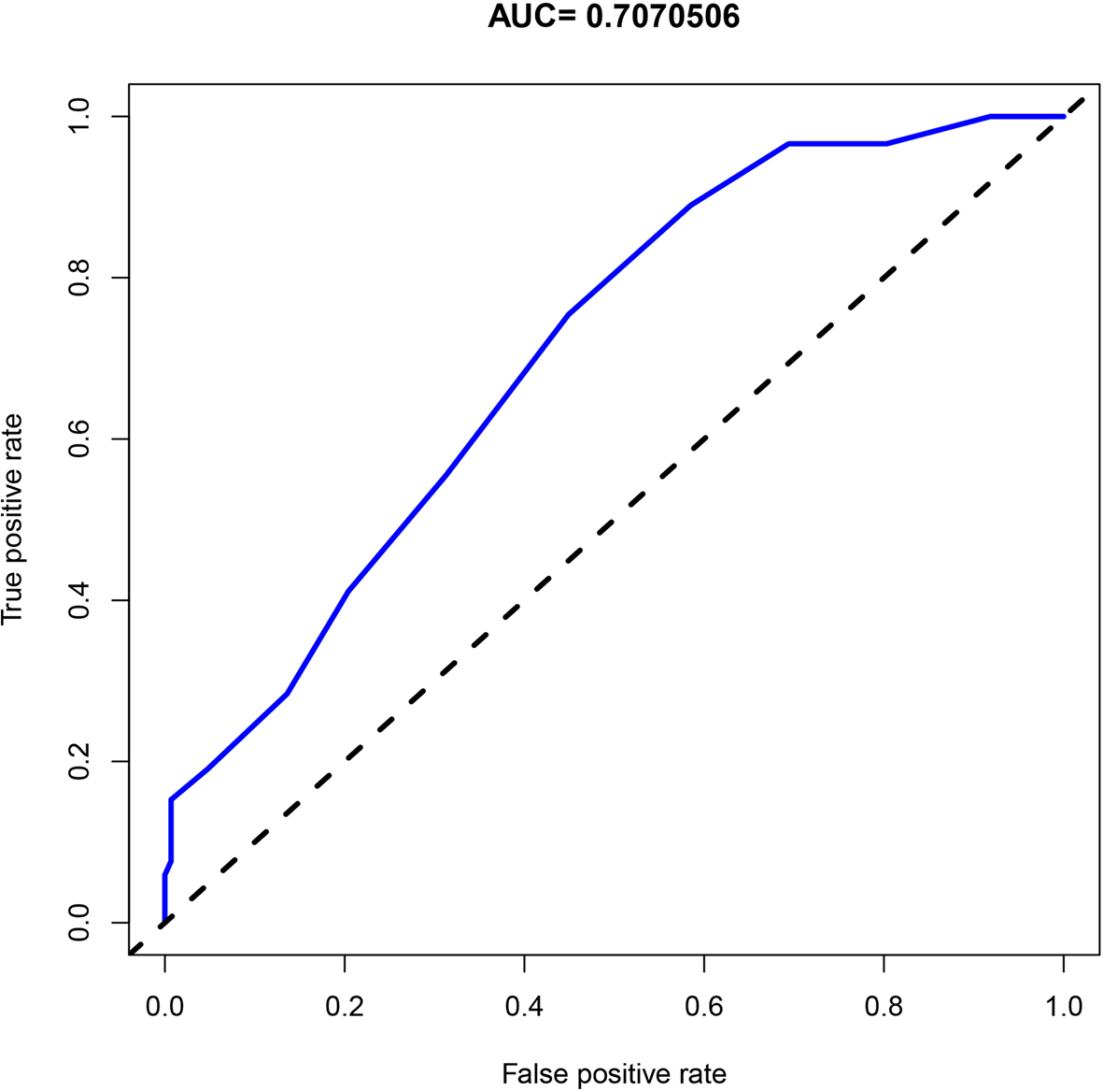
The AUC value for ALB/GLB of validation set

After ML, ALB/GLB ratio was selected as one of the factors for the final HLA-B27 diagnostic model (Fig. 5). As can be seen from Fig. 6a, when ALB/GLB ratio alone was used as a diagnostic factor for training set, with its AUC value being 0.756. The C-value of the ALB/GLB ratio was computed as 0.756. In the validation cohort, ALB/GLB ratio achieved an AUC value of 0.7070506 (Fig. 9), and the C-value was 0.707. In patients with AS, the ALB/GLB ratio among cases with HLA-B27 positivity was considerably lower in relative to those with HLA-B27 negativity.

### Differences between patients with HLA-B27 positivity and those with HLA-B27 negativity undergoing AS

There existed no obvious difference in sex and age between cases with HLA-B27 positivity and those with negativity (Table 5). Additionally, mean BASFI and BASDAI scores of cases with HLA-B27 positivity were higher compared with cases with HLA-B27 negativity, but there existed no statistical difference. As can be seen from Table 6, the duration of morning stiffness among cases with HLA-B27 negativity was mainly around 1 h, whereas that among cases with HLA-B27 positivity was mainly concentrated in those without morning stiffness, and the difference between the two showed statistical significance. The degree of night pain did not differ significantly between the two cohorts.

**Table 5:** Difference between AS cases showing HLA-B27 positivity and negativity

	**HLA-B27 positivity (*N* = 124)**	**HLA-B27 negativity (*N* = 24)**	***P*-value**
**Sex**			
Male	108 (87.1%)	20 (83.3%)	0.622
Female	16 (12.9%)	4 (16.7%)	
**Age**			
Mean (SD)	34.35 (9.78)	33.54 (13.63)	0.728
Media 【Min, MAX】	33.5 【16,63】	32.5 【9,66】	
**ALB/GLB**			
Mean (SD)	1.31 (0.337)	1.69 (0.462)	< 0.001
Median 【Min, MAX】	1.3 【0.7,2.2】	1.7 【1,3.2】	
**BASFI**			
Mean (SD)	26.3 (20.4)	22.5 (21.2)	0.411
Median【Min, MAX】	15 【6,92】	15 【6,92】	
**BASDAI**			
Mean (SD)	1.97 (1.57)	1.4 (1.16)	0.097
Median 【Min, MAX】	1.05 【0,7.1】	0.9 【0,4.7】

**Table 6:** Difference between AS cases with HLA-B27 positivity and negativity

	**HLA-B27 positivity** **(*N* = 124)**	**HLA-B27 negativity** **(*N* = 24)**	***P*-value**
**The pain at night**			
No symptom	64 (51.6%)	13 (54.1%)	0.99
Mild pain	48 (38.7%)	9 (37.5%)	
Moderate pain	7 (5.7%)	1 (4.2%)	
Severe pain	5 (4%)	1 (4.2%)	
**Morning stiffness time**			
No symptom	76 (61.3%)	8 (33.3%)	0.024
One hour	33 (26.6%)	14 (58.3%)	
Two hours	9 (7.3%)	1 (4.2%)	
Three hours	6 (4.8%)	1 (4.2%)	

## Discussion

In this study, a predictive model for HLA-B27 diagnosis was established using an ML algorithm based on clinically relevant data. The prediction model is on the basis of various estimations [25]. Meanwhile, we employed 3 ML models for filtering variables. Moreover, clinicians may use this AI-based strategy with the aim of better diagnosing diseases and analyzing the differences between cases with HLA-B27 positivity and those with HLA-B27 negativity [26]. To explore the difference of symptoms between cases with HLA-B27 positivity and those with negativity, we used the data of patients with AS.

ML has contributed immensely to the healthcare paradigm change, and one of its biggest advantages in healthcare is that the performance can be continuously enhanced in accordance with updates by automatically learning from data [27]. Thus, ML provides the merits of strong ability, objectivity, as well as repeatability when handing large datasets and reliable data [28]. Apart from prompting the clinician–patient sharing of information about decision-making, these benefits also allow the planning to be effective and the healthcare utilization to be reasonable. ML is able to enhance the quality of early diagnosis and detetct disease progression [29].

HLA-B27 is often used to aid in the diagnosis of immunological diseases, especially in patients with AS [30]. We searched for differences in 1503 patients who had undergone the HLA-B27 test and established the prediction model for RBC, MCHC, MPV, ALB/GLB ratio, PAB, and HCO.

RBC counts were reduced in HLA-B27-negative cases from both sets; no such observations have been reported in other studies. Ninety percent of RBCs consist of HGB, which functions to transport carbon dioxide (CO2) and oxygen. RBCs also act as immunoglobulin carriers, lower T cell proliferation, and stimulate phagocytosis. In addition, HLA-B27 is mainly responsible for delivering endogenous peptides (primarily T-cell antigen receptors (TCR)) into T lymphocytes (cytotoxic T-cells) [6]. Whether the reduction in T cell proliferation by RBCs has an impact on HLA-B27 in the human body needs to be further explored.

The average MCHC is decreased in HLA-B27 positive patients in both the training and validation cohorts. Common causes of reduced MCHC include hemodilution, anemia, and hematological diseases [31]. Hadi et al. found that supplementation of betaine, which regulates immune function, can significantly improve MCHC in the human body [32]. We hypothesize that the impact of HLA-B27 on the immune system was responsible for the decrease in MCHC among cases with HLA-B27 positivity. HCO elevated among cases with HLA-B27 positivity. High HCO in renal function is more common in clinical alkali poisoning, whereas mild HCO elevation without other obvious symptoms has no significant clinical significance. Further studies are required to demonstrate the practical significance of HCO elevation among cases with HLA-B27 positivity.

MPV considerably decreased among cases with HLA-B27 positivity relative to those with HLA-B27 negativity; however, the mean BPC increased compared with normal among HLA-B27-positive patients. In research by Liang et al., platelets in patients undergoing AS remarkably increased than normal subjects [33]; normal platelet range was 100–300 × 10^9^/L. During the development of platelets, the size of platelets varies, and the volume of platelets gradually decreases from developing high-quality platelets to aging platelets. HLA-B27-positive patients had lower MPV, and most platelets were considered aging, or it could be related to splenic function. Platelets are mainly involved in blood clotting in humans, and the activation of blood clotting is associated with axial spinal arthritis [34]. In the current study, we did not collect data regarding the coagulation function of patients receiving HLA-B27 for the examination; thus, further study is required.

ALB was low among cases with HLA-B27 positivity in the high GLB level; thus, ALB/GLB ratio was significantly reduced among cases with HLA-B27 positivity. Data collected from cases with AS revealed a low ALB/GLB ratio among cases with HLA-B27 positivity (Table 6). The ALB/GLB ratio holds significant clinical significance. Firstly, it serves as an indicator for evaluating patients' nutritional status. Secondly, both ALB and GLB are proteins synthesized by the liver, meaning their levels can indicate liver dysfunction. Elevated GLB levels can be seen in conditions such as chronic liver disease or inflammation, while a decreased ALB/GLB ratio may suggest impaired liver function. The ALB/GLB ratio has also been found to be related to disease prognosis in certain cases. For instance, a lower ALB/GLB ratio is associated with poorer outcomes in patients with certain cancers or chronic kidney diseases. The nutritional status of HLA-B27 positive patients is often poor. HLA-B27 positive patients are often plagued by AS or other immune diseases, which deplete the body nutrients for a long time, thus leading to the reduction of ALB. GLB is produced by the immune system and is engaged in human immunity. Liu et al. discovered that ALB of patients with AS obviously decreased relative to normal group, whereas GLB increased compared with normal group [34]. ALB/GLB ratio may become a new research direction for immune diseases in the future. PAB is synthesized by hepatocytes and has a very short half-life. It is a sensitive nutritional protein index. PAB is often decreased in acute inflammation, tumor, liver cirrhosis and other diseases [35]. PAB was low in HLA-B27 positive patients, as was ALB, again indicating poor nutritional status.

BASFI, BASDAI, night pain, and morning stiffness were compared between cases showing HLA-B27 positivity and negativity with AS. Activity scores among cases with HLA-B27 positivity increased relative to those with HLA-B27 negativity, indicating less restriction among cases with HLA-B27 positivity relative to HLA-B27 negativity, which was also confirmed by Marta et al. [36]. Misfolding of HLA-B27 in AS mice clearly leads to the clinical manifestations of arthritis and elevated inflammatory mediators in mice [37]. There have also been studies on the familial aggregation and earlier onset of AS patients with HLA-B27 positive surface [38]. There existed no obvious difference in night pain between the two cohorts, and the duration of morning stiffness among cases with HLA-B27 negativity increased relative to those showing HLA-B27 positivity. However, the sample size was small, thus necessitating confirmation by further study.

There are various ways to use ML to process clinical data. For example, multiple diagnostic models can be constructed through a variety of ML and the diagnostic performance of no single diagnostic model can be compared to screen out the best diagnostic model [39, 40]. In this study, a dataset of 1503 patients was used to help choose the best ML prediction. This study offers several advantages. First, few studies have examined HLA-B27-positive patients by using multiple factors, including sex, age, ESR, CRP, liver/kidney function tests and routine blood examination. Second, three ML methods were adopted for filtering the data and the validation cohort was employed for validation. Third, we investigated the differences between AS cases with 148 HLA-B27 positivity and those with negativity. Finally, the proposed model showed good predictive ability, which is helpful for clinicians to infer the results of HLA-B27, which can be used to help diagnose related diseases.

Nevertheless, there are certain limitations of the current work. First, the retrospective nature of the study may generate selection and subjective biases. Second, the as-proposed ML algorithm model was only developed in a hospital. Moreover, it can probably restrict the application in additional areas and require further verification. Thirdly, this study only focused on the blood differences of patients with different results of HLA-B27, and did not further study other differences. Fourthly, there is a certain difference between the positive and negative ratios of the training set and the validation set before, which is the error caused by random sampling, which can easily lead to false responses and affect our results. Furthermore, due to the modification in the hospital's inpatient case system, we are only able to collect detailed inpatient data from 2018 onwards. This differs from the duration of data collection for HLA-B27, which may impact the accuracy of the study. Finally, due to the performance of our computer, ML cannot be applied to analyze all data from the beginning, which may lead to some differences in the selected variables.

## Conclusion

To conclude, in the current work, we attempted to construct the prediction model of HLA-B27. The overall diagnostic performance of the model is satisfactory, and doctors can accurately infer the results of HLA-B27 using our nomogram and thereby aiding doctors in diagnosing a variety of immune diseases. We also found that ALB/GLB in AS and other immune diseases may become a new research hotspot in the future. Certainly, clinicians usually interpret results by using the corresponding domain expertise. Moreover, it is expected that in the near future, the diagnostic model proposed in this study will also include the broad scope of clinical variables, and thus it can be applied more to a wider population.

### Supplementary Information

**Additional file 1.**

## Data Availability

Article display data used in this work. The datasets generated during the current study are not publicly available due. For further inquiries, please contact the corresponding authors.
