# Supplementary material for: Development and validation of a machine learning-based nomogram for predicting HLA-B27 expression
Source: BMC Immunol. 2023 Sep 26;24:32. doi: 10.1186/s12865-023-00566-z (PMC10521518; doi:10.1186/s12865-023-00566-z)
Supplement: Supplementary file 1 — Additional file 1. [file 12865_2023_566_MOESM1_ESM.pdf]

广西医科大学第一附属医院
论文伦理审批意见
FIRST AFFILIATED HOSPITAL of GUANGXI MEDICAL UNIVERSITY
ETHICAL REVIEW COMMITTEE Approval Notice

申请人<br>Applicant	刘冲	申请日期<br>Date of Application	2022-06-29
编号<br>Approval Number	NO.2022-KY-E-(211)	所属学科<br>Discipline	脊柱骨病外科
题目<br>Title	Development and validation of a machine learning-based nomogram for prediction of HLA-B27 expression		
研究内容<br>Research Contents	We found that many regional hospitals lack the testing equipment for HLA-B27, which affects the diagnosis. We aimed to determine the differences by collecting clinical data related to HLA-B27 test patients and establish a diagnostic model to infer the results of HLA-B27 by using ML methods to help clinicians better diagnose diseases. We also examined the differences in symptoms between HLA-B27-positive and negative patients with AS.		
附件	HLA-B27论文伦理审核表.pdf<br>填写说明.doc		
依托项目<br>Acknowledgement	国家自然科学基金		
其他			
伦理委员会办公室主任意见<br>Director of ethics office	同意<br>同意<br>(2022-06-29 16:22)		
（副）主任委员意见<br>(Vice)Director of Ethical Review Committee			
结 论<br>Conclusion	This project fully considered and protected the rights and interests of the study objects. It meets the criteria of Ethical Review Committee. The Medical Ethics Committee of First Affiliated Hospital of Guangxi Medical University has approved the protocol.		
生效日期<br>Date of Approval	2022-06-29		

广西医科大学第一附属医院医学伦理委员会
FIRST AFFILIATED HOSPITAL of GUANGXI MEDICAL UNIVERSITY
ETHICAL REVIEW COMMITTEE

地址：中国广西南宁市双拥路6号，邮编530021
联系电话：0771-5356557，传真：0771-5359801，邮箱：gxmuyfyll@163.com
NO.6, Shuangyong Road, Nanning, Guangxi, P.R.China, 530021
Tel: +86 771 5356557 Fax: +86 771 5359801 E-mail: gxmuyfyll@163.com

扫描全能王 创建
